# Lung function trajectories from school age to adulthood and their relationship with markers of cardiovascular disease risk

**DOI:** 10.1136/thorax-2023-220485

**Published:** 2024-05-02

**Authors:** Raquel Granell, Sadia Haider, Matea Deliu, Anhar Ullah, Osama Mahmoud, Sara Fontanella, Lesley Lowe, Angela Simpson, James William Dodd, Seyed Hasan Arshad, Clare S Murray, Graham Roberts, Alun Hughes, Chloe Park, John W Holloway, Adnan Custovic

**Affiliations:** 1 Department of Population Health Sciences, Bristol Medical School, University of Bristol, Bristol, UK; 2 National Heart and Lung Institute, Imperial College London, London, UK; 3 Mathematical Sciences, University of Essex, Colchester, UK; 4 Applied Statistics, Helwan University Faculty of Commerce, Cairo, Egypt; 5 Division of Infection, Immunity and Respiratory Medicine, School of Biological Sciences, Faculty of Biology, Medicine and Health, University of Manchester, Manchester, UK; 6 Academic Respiratory Unit, North Bristol NHS Trust, Westbury on Trym, UK; 7 MRC Integrative Epidemiology Unit, Bristol, UK; 8 Allergy, The David Hide Asthma & Allergy Research Centre, Newport, UK; 9 Respiratory Group, University of Manchester, School of Translational Medicine, Manchester, UK; 10 Human Development and Health Academic Unit, University of Southampton Faculty of Medicine, Southampton, UK; 11 Respiratory Biomedical Research Unit, Southampton University Hospitals Trust, Southampton, UK; 12 MRC Unit for Lifelong Health and Ageing at UCL, Department of Population Science & Experimental Medicine, Institute of Cardiovascular Science, UCL, London, UK

**Keywords:** Respiratory Measurement

## Abstract

**Rationale:**

Lung function in early adulthood is associated with subsequent adverse health outcomes.

**Objectives:**

To ascertain whether stable and reproducible lung function trajectories can be derived in different populations and investigate their association with objective measures of cardiovascular structure and function.

**Methods:**

Using latent profile modelling, we studied three population-based birth cohorts with repeat spirometry data from childhood into early adulthood to identify trajectories of forced expiratory volume in 1 s (FEV_1_)/forced vital capacity (FVC). We used multinomial logistic regression models to investigate early-life predictors of the derived trajectories. We then ascertained the extent of the association between the derived FEV_1_/FVC trajectories and blood pressure and echocardiographic markers of increased cardiovascular risk and stroke in ~3200 participants at age 24 years in one of our cohorts.

**Results:**

We identified four FEV_1_/FVC trajectories with strikingly similar latent profiles across cohorts (pooled N=6377): above average (49.5%); average (38.3%); below average (10.6%); and persistently low (1.7%). Male sex, wheeze, asthma diagnosis/medication and allergic sensitisation were associated with trajectories with diminished lung function in all cohorts. We found evidence of an increase in cardiovascular risk markers ascertained by echocardiography (including left ventricular mass indexed to height and carotid intima-media thickness) with decreasing FEV_1_/FVC (with p values for the mean crude effects per-trajectory ranging from 0.10 to p<0.001). In this analysis, we considered trajectories as a pseudo-continuous variable; we confirmed the assumption of linearity in all the regression models.

**Conclusions:**

Childhood lung function trajectories may serve as predictors in the development of not only future lung disease, but also the cardiovascular disease and multimorbidity in adulthood.

**WHAT IS ALREADY KNOWN ON THIS TOPIC**

- In utero and early-life factors have been shown to influence lung function trajectory through childhood and can influence the lung function attained at the physiological peak in early adulthood.

**WHAT THIS STUDY ADDS**

- Little is known about the relationship between lung function development during childhood and preclinical markers of cardiovascular and metabolic disease risk. We ascertained the association of lung function trajectories from childhood to early adulthood derived using data-driven methods with objective measures of cardiovascular structure and function ascertained using echocardiogram data and carotid artery scans (which are markers of preclinical cardiovascular risk and can predict subsequent cardiovascular disease).

**HOW THIS STUDY MIGHT AFFECT RESEARCH, PRACTICE OR POLICY**

- Our study highlights the importance of lung growth and its association with adverse respiratory, cardiovascular and metabolic outcomes, and the importance of identifying early life risk factors. Our findings draw attention to the potential importance of measuring lung function from early school age as a marker of future risk, since early lung function optimisation to alter trajectories may help in preventing adverse health outcomes in adulthood.

## Introduction

Spirometry is the most commonly used pulmonary function test for identifying patterns of physiological abnormalities. Spirometric impairments (both airflow obstruction and restrictive ventilatory defect) are related to adverse health outcomes.^1^ For example, diminished forced expiratory volume in 1 s (FEV_1_)/forced vital capacity (FVC), which is a hallmark of chronic obstructive pulmonary disease (COPD), is also associated with cardiovascular morbidity and mortality.^2^ Low FEV_1_ is associated with contemporaneous cardiovascular disease in adults, and similar relationships have been observed for FVC.

In recent years, a substantial effort has been devoted to identifying lifetime lung function trajectories based on different spirometry measures and their associations with early-life risk factors and subsequent health outcomes (reviewed in Okyere *et al* 3). Potential implementation of this knowledge in clinical practice to detect poor lung health early is attracting increasing attention.^4^ Due to limited availability of repeated spirometry measurement in children, relatively few studies extended the modelling of trajectories to childhood lung function (online supplemental table S1).^3^ In such studies, in utero and early-life factors have been shown to influence trajectory through childhood and have an important impact on the lung function attained at the physiological peak in early adulthood. Early life factors associated with diminished lung function in early adulthood include preterm birth, respiratory infections, allergic sensitisation, childhood asthma and persistent wheezing, and exposure to tobacco smoke in utero. Poor intrauterine growth and nutritional deficits during pregnancy and childhood precede and predict the development of spirometric restriction in adulthood.^5 6^ Importantly, diminished lung function at physiological peak is an independent marker of not only respiratory disease in later adulthood, but also cardiovascular morbidity and early all-cause mortality.^1 7–9^ However, little is known about the relationship between lung function development during childhood and preclinical markers of cardiovascular and metabolic disease risk, and whether assessment of spirometry in childhood may be informative about the future cardio-metabolic health.

10.1136/thorax-2023-220485.supp1 Supplementary data

We hypothesise that diminished childhood lung function trajectories are associated with preclinical markers of cardiovascular and metabolic disease. To address our hypothesis, we first modelled lung function from early school age to physiological peak in the third decade in three UK birth cohorts with repeated spirometry through childhood to ascertain whether stable and reproducible trajectories can be derived in different populations. We focused on modelling FEV_1_/FVC as a marker of airway obstruction to facilitate comparison of our findings with previous studies such as TCRS,^10^ PELOTAS^11^ and RAINE.^12^ We then capitalised on the availability of the ultrasound scans of carotid arteries and echocardiograms including carotid artery intima-media thickness (cIMT) and the measurement of pulse wave velocity (PWV), (blood pressure and blood triglycerides) at age 24 years in subjects in the Avon Longitudinal Study of Parents and Children (ALSPAC) without overt clinical cardiovascular and metabolic disease.^13^ We ascertained the association of the derived lung function trajectories with these objective measures of cardiovascular structure and function, which are markers of preclinical cardiovascular risk and which predict subsequent cardiovascular disease.^14–16^

## Methods

Detailed description of cohorts, methods and analyses is presented in online supplemental file.

### Study design, setting and participants

We used data from three UK population-based birth cohorts in the STELAR/UNICORN consortium: ALSPAC,^17^ Isle of Wight (IOW)^18^ and Manchester Asthma and Allergy Study (MAAS).^19^ Data were integrated in a web-based knowledge management platform to facilitate joint analyses.^20^

### Data sources/measurements

Spirometry was available at ages 8, 11, 16 and 20 years in MAAS; 8, 15 and 24 years in ALSPAC; and 10, 18 and 26 years in IOW. Details of clinical follow-up and definitions of outcomes including asthma, wheeze phenotypes from birth to early adulthood,^21^ severe asthma exacerbations, lower respiratory tract infections (LRTIs) and environmental exposures are provided in online supplemental file.

### Assessment of cardiovascular risk in ALSPAC

Left ventricular (LV) mass indexed to height^2.7^ (LVMI, g/m^2.7^), LV posterior wall (PW) systolic thickness average (LVPW, cm), carotid femoral PWV (m/s), pulse pressure (mm Hg), average cIMT mean (mm), systolic and diastolic blood pressure (BP) (mm Hg), triglycerides (log-transformed) and high-density lipoprotein (HDL, mmol/L) were measured at research clinics at age 25 years.^13^

Ultrasound scans of the left and right common carotid arteries were performed using a CardioHealth Panasonic system with a 13–5 MHz linear array broadband transducer according to a standardised protocol to measure cIMT. Echocardiography was performed using a Philips EPIQ 7G Ultrasound in accordance with American Society of Echocardiography guidelines. PWV was measured using a Vicorder device validated in adolescents.^22^ Three PWV measurements were taken with an interval of 1 min between measurements, acceptable PWV measurements were within ≤0.5 m/s of each other. Results were averaged to give a measurement of arterial stiffness. In MAAS, blood pressure was measured at age 20 years.

### Statistical analysis

We used latent profile modelling to derive trajectory classes based on the development of FEV_1_/FVC over time in three cohorts independently. We analysed data from participants who had spirometry on at least two occasions under the assumption that data were missing at random. Briefly, we used two-level random intercept regression models to assign children to their most likely trajectory profile. The models were compared for goodness-of-fit using the Bayesian Information Criterion (BIC). For each child, the posterior probability of belonging to each of the classes was estimated, and children were classified to each trajectory profile based on their maximum posterior probability.

All analyses were repeated for those with complete spirometry data to test the sensitivity and confirm robustness of the derived trajectories.

We used weighted multinomial logistic regression models to ascertain early-life risk factors associated with each lung function trajectory. The posterior probability of membership for each trajectory class was used as weights to reflect uncertainty of class assignment; results are reported as relative risk ratios (RRR) with 95% CIs.

We used linear regression models to assess the associations between lung function trajectories between 8 and 24 years and markers of cardiovascular and metabolic disease risk at 24 years. We report both individual trajectory effects and per-trajectory effects; in this analysis, we considered trajectories as a pseudo-continuous variable; we confirmed the assumption of linearity in all the regression models. All models were weighted by class membership probabilities. Additionally, we performed sex-stratified analyses and further adjustment by low birth weight and tobacco smoke exposure. When considering complete cases in both crude and adjusted analyses, the persistent low trajectory was most affected, with numbers of individuals dropping from 80 to as low as 20–22 in the associations with cardiovascular outcomes.

## Results

We included 4874 participants from ALSPAC, 809 from IOW and 801 from MAAS, who had completed spirometry on at least two occasions during the follow-up. Characteristics of the study populations and comparisons between subjects included and excluded from the analyses are shown in online supplemental table S2. In MAAS and IOW, there was a lower prevalence of parental smoking at recruitment and a higher prevalence of breast feeding among included participants. There was a lower proportion of males in the analysed sample in IOW.

### FEV_1_/FVC trajectories from early school-age to young adulthood

**Figure 1:**
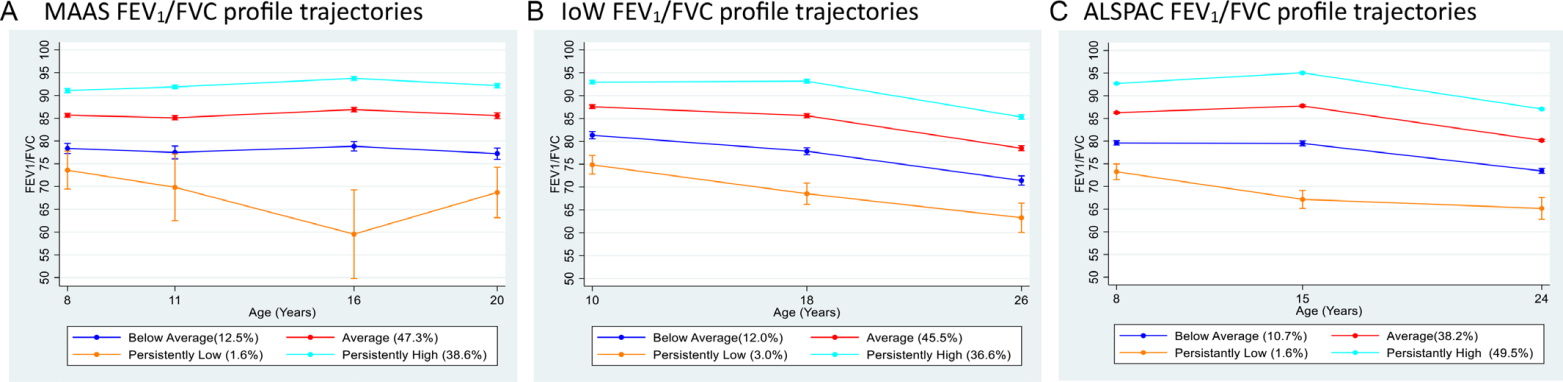
Mean FEV_1_/FVC over time in the four trajectory classes. ALSPAC, Avon Longitudinal Study of Parents and Children; FEV_1_, forced expiratory volume in 1 s; FVC, forced vital capacity; IOW, Isle of Wight; MAAS, Manchester Asthma and Allergy Study.

The best-fitting model was selected as four FEV_1_/FVC trajectories in all three cohorts (online supplemental table S3 and figure 1). BIC was marginally lower for the 5-class model for MAAS, but we opted for a more parsimonious solution. Based on the developmental pattern of FEV_1_/FVC, these trajectories were labelled as: (1) above average; (2) average; (3) below average; and (4) persistently low (figure 1). Study participants within the four trajectories had stable lung function that tracked from early school age to adulthood, with no overlap in FEV_1_/FVC between the trajectories at any time (figure 1A–C). The highest within-class variability in the individual FEV_1_/FVC trajectories was observed in the persistently low trajectory. Importantly, the proportion of allocated participants and the mean FEV_1_/FVC values over time in each of the trajectories were consistent across the cohorts (table 1).

**Table 1:** The assigned number of children and the mean FEV_1_/FVC during the follow-up (with 95% CI) per FEV_1_/FVC trajectory

Trajectories 8–26 years	MAAS		IOW		ALSPAC	
	N (%)	FEV_1_/FVC	N (%)	FEV_1_/FVC	N (%)	FEV_1_/FVC
		Mean (95% CI)		Mean (95% CI)		Mean (95% CI)
Above average	309	92.21	320	91.02	2355	91.81
	38.60%	(91.98 to 92.44)	39.60%	(90.68 to 91.37)	49.40%	(91.68 to 91.94)
Average	379	85.78	368	84.61	1816	85.06
	47.30%	(85.53 to 86.03)	45.50%	(84.26 to 84.97)	38.10%	(84.91 to 85.21)
Below average	100	78.01	97	77.53	516	77.94
	12.50%	(77.40 to 78.62)	12.00%	(76.85 to 78.21)	10.80%	(77.61 to 78.27)
Persistently low	13	67.77	24	69.76	80	69.11
	1.60%	(64.28 to 71.35)	3.00%	(67.98 to 71.54)	1.70%	(67.88 to 70.34)

ALSPAC, Avon Longitudinal Study of Pregnancy and Childhood; FEV_1_, forced expiratory volume in 1 s; FVC, forced vital capacity; IOW, Isle of Wight; MAAS, Manchester Asthma and Allergy Study.

The posterior probability of class membership was high in all cohorts (>0.7), indicating high confidence in class assignment (online supplemental table S4). Class assignments were robust to the presence of missing data, with the proportion of children assigned to the same class in samples with complete and >2 observations exceeding 75% (online supplemental table S5).

### Sex, demographic and environmental characteristics of FEV_1_/FVC trajectories

Online supplemental table S6 shows results of multinomial logistic regression models weighted for the probability of each individual belonging to each trajectory, using the average class as the reference. Males had a higher risk of being in the persistently low trajectory (MAAS and ALSPAC). Low birth weight was associated with persistently low trajectory in ALSPAC (RRR 2.30, 95% CI 1.05 to 5.06, p=0.038). Maternal smoking during pregnancy and/or the child’s first year of life increased the risk of below average (1.30, 1.01 to 1.67, p=0.04) and persistently low (1.60, 0.93 to 2.76, p=0.09) trajectories in ALSPAC, with similar estimates in IOW. Paternal asthma increased the risk of below average in MAAS (2.02, 1.14 to 3.59, p=0.017) and maternal asthma increased the risk of persistently low in ALSPAC (1.88, 1.07 to 3.30, p=0.027). Increasing preschool age body mass index (BMI) was associated with increased risk of below average trajectory in MAAS (1.23, 1.09 to 1.4, p=0.001), while decreasing childhood BMI increased the risk of above average trajectory in MAAS (0.95, 0.89 to 1.02, p=0.03) and ALSPAC (0.92, 0.89 to 0.95, p=1.11E-06), with a similar trend in IOW.

### Association between FEV_1_/FVC trajectories, asthma diagnosis, wheeze and sensitisation

The persistently low FEV_1_/FVC trajectory was associated with current wheeze and current asthma diagnosis in all cohorts (online supplemental table S7). For example, current asthma in ALSPAC was strongly associated with persistently low trajectory (RRR 3.62, 95% CI 2.14 to 6.11, p=1.5×10^−6^), and in general, the likelihood of asthma diagnosis increased with decreasing trajectory.

We capitalised on the availability of data from healthcare records in MAAS to show evidence of an association between diminished lung function trajectories and LRTIs and asthma/wheeze hospital admissions by age 3 years, with markedly increased risks for below average and persistently low trajectories (online supplemental table S7). Respiratory syncytial virus-confirmed bronchiolitis was one of the strongest associates of persistently low trajectory (RRR 6.7, 95% CI 1.30 to 34.88, p=0.023).

We found strong evidence of an association between trajectory membership and allergic sensitisation in all cohorts. Sensitisation in preschool and early school age increased the risk of membership of the persistently low trajectory (ALSPAC age 7, RRR 2.41, 95% CI 1.44 to 4.03, p<0.0001; IOW age 4, RRR 3.84, 95% CI 1.44 to 10.21, p=0.007). In MAAS, children in the Above average lung function trajectory were less likely to be sensitised after age 3 years.

### Association between FEV_1_/FVC trajectories and wheeze phenotypes

**Figure 2:**
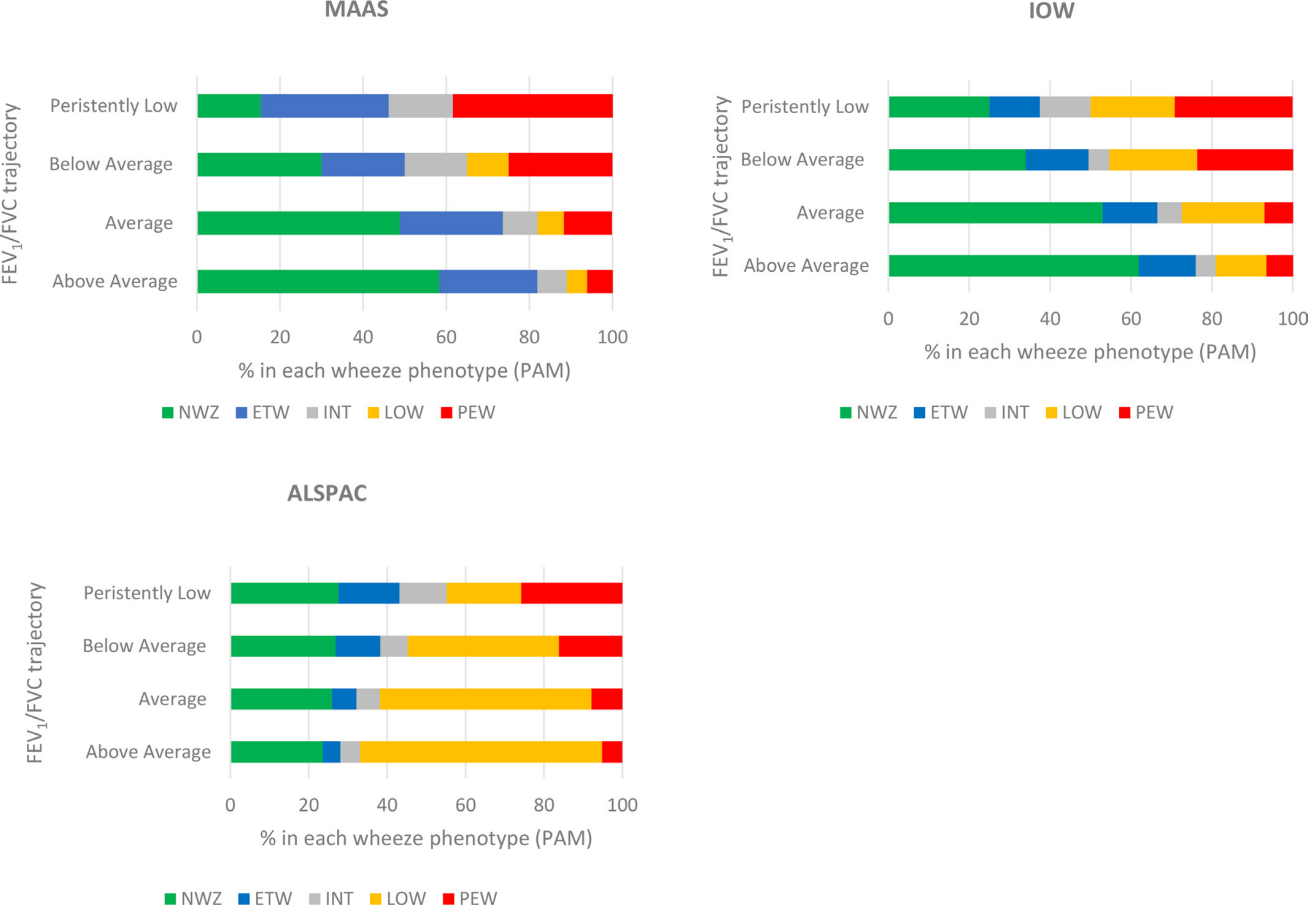
Distribution of partition-around-medoids (PAM) wheeze phenotypes (ETW, INT, LOW, NWZ, PEW) membership^21^ by FEV_1_/FVC assigned classes. ALSPAC, Avon Longitudinal Study of Parents and Children; ETW, early-transient; FEV_1_, forced expiratory volume in 1 s; FVC, forced vital capacity; INT, intermittent; IOW, Isle of Wight; LOW, late-onset; MAAS, Manchester Asthma and Allergy Study; NWZ, never; PEW, persistent wheeze.

The proportion of participants in the persistent wheeze cluster increased with decreasing lung function trajectory, although it is of note that 5%–6% of those in the above average trajectory had persistent wheeze (figure 2, online supplemental table S8).^21^

### Lung function trajectories and cardiovascular and metabolic outcomes in ALSPAC

Table 2 shows the crude and adjusted risk of cardiovascular outcomes at 24 years per-FEV_1_/FVC trajectory increase (ie, with decreasing lung function). These analyses were performed on 1422–2759 individuals with data available in ALSPAC.

**Table 2:** Associations between FEV_1_/FVC trajectories (8–24 years) and markers of cardiovascular disease risk at 24 years in 1700–3200 individuals in Avon Longitudinal Study of Parents and Children

	N	Mean 95% CI Per-FEV_1_/FVC trajectory 8–24 years Crude effect	P value	Mean 95% CI Per-FEV_1_/FVC trajectory 8–24 years Adjusted effect†	P value	Attenuating confounders
Cardiovascular outcomes at 24 years						
Left ventricular mass indexed to height 2.7 (g/m^2.7^)	1460	1.14 (0.68 to 1.60)	<0.001	0.58 (0.14 to 1.02)	0.009	Sex and BMI
Left ventricle posterior wall systolic thickness average (cm)	1422	0.033 (0.020 to 0.045)	<0.001	0.014 (0.002 to 0.025)	0.02	Sex
Carotid femoral pulse wave velocity (m/s)	1702	0.058 (−0.011 to 0.13)	0.10	−0.012 (−0.079 to 0.056)	0.73	Sex
Pulse pressure (mm Hg)	2759	1.10 (0.65 to 1.55)	<0.001	0.005 (−0.38 to 0.39)	0.98	Sex
Average carotid intima-media thickness-mean (mm)	1451	0.005 (0.001 to 0.008)	0.008	0.003 (−0.001 to 0.006)	0.12	Sex and BMI
Blood pressure measures at 24 years						
Systolic (mm Hg)	2759	1.44 (0.87 to 2.02)	<0.001	0.07 (−0.43 to 0.58)	0.77	Sex
Diastolic (mm Hg)	2759	0.34 (−0.06 to 0.74)	0.09	0.07 (−0.33 to 0.47)	0.73	Sex, BMI
Fasting lipids at 24 years						
Triglycerides (mmol/L, log)	2269	0.032 (0.009 to 0.056)	0.006	0.016 (−0.007 to 0.039)	0.18	Sex, BMI
HDL (mmol/L)	2269	−0.035 (−0.059 to –0.012)	0.003	−0.006 (−0.029 to 0.016)	0.58	Sex

Lung function trajectories treated as continuous: (1) above average (49.5%); (2) average (38.3%); (3) below average (10.6%); and (4) persistently low (1.7%). Linear regression crude and adjusted analyses weighted by class membership probabilities. We tested the assumption of linearity in all the regression models using lrtest command in Stata. P values from likelihood ratio tests were ≥0.05.

*Adjusted by sex, maternal lower education level (educated to the General Certificate of Education level ‘school-leaving certificate’ or lower) and child’s BMI at 7 years. Note: ‘per-class increase’ is equivalent to ‘with decreasing lung function’.

BMI, body mass index; FEV_1_, forced expiratory volume in 1 s; FVC, forced vital capacity; HDL, high-density lipoprotein.

We found an increase in LVMI (mean 1.14 g/m^2.7^, 95% CI 0.68 to 1.60 per FEV_1_/FVC trajectory; p=1.30×10^−6^), increase in LVPW systolic thickness average (mean 0.03 cm, 95% CI 0.02 to 0.05; p=2.0×10^−7^), increase in average cIMT mean (mean 0.005 mm, 95% CI 0.001 to 0.008; p=0.008) and increase in pulse pressure (mean 1.10 mm Hg, 95% CI 0.65 to 1.55; p=1.82×10^−6^), with decreasing lung function. Furthermore, we observed an increase in systolic BP (mean 1.44 mm Hg, 95% CI 0.87 to 2.02 per-lung function trajectory; p=8.4×10^−7^), higher serum triglycerides (mean 0.03 mmol/L, 95% CI 0.01 to 0.06 per-lung function trajectory; p=0.006), and lower HDL (mean −0.04 mmol/L, 95% CI −0.06 to −0.01, p=0.003), with decreasing lung function. After adjustment by sex, maternal education level and child’s BMI, the only remaining associations were for LVMI (mean 0.58 g/m^2.7^, p=0.009) and LVPW systolic thickness average (mean 0.01 cm, p=0.02); with small evidence for residual associations for average cIMT mean.

We observed similar effect in relation to systolic BP in MAAS, with an increase in BP with decreasing lung function (mean 2.05 mm Hg, 95% CI 0.63 to 3.47 per trajectory; p=0.005) (online supplemental table S9). This difference was completely attenuated after adjustment for sex and BMI.

Results for individual effects for each lung function trajectory (average as reference category) are reported in online supplemental tables S10–S12. Sex-stratified analyses (online supplemental table S13) show similar effects for LVMI (males: mean 0.79 g/m^2.7^ per-lung function trajectory, p=0.03; females: mean 0.59 g/m^2.7^, p=0.06) but an attenuation of the effect for LVPW systolic thickness average in females (males: mean 0.02 cm, p=0.08; females: mean 0.01 cm, p=0.17).

Online supplemental table S14 compares associations using predicted FEV_1_/FVC trajectories (which are adjusted by age, ethnicity, height and gender) vs raw FEV_1_/FVC adjusted by sex, maternal education level and child’s BMI in ALSPAC. While some of the associations between trajectories and CV markers failed to reach formal statistical significance, this was not the case for all of them (associations with LVMI and LVPW, with a trend for cIMT remained significant).

## Discussion

In this study, we used data-driven analyses in three independent birth cohorts to identify four FEV_1_/FVC trajectories extending from early school age into early adulthood (above average, average, below average and persistently low). Results were highly consistent across the populations, with no overlap in FEV_1_/FVC at any follow-up time in all cohorts. Membership of the persistently low trajectory was associated with male sex, wheeze and allergic sensitisation in all cohorts. Individuals assigned to the persistently low trajectory were at an increased risk of having an asthma diagnosis through childhood, adolescence and early adulthood. Decreasing BMI in preschool and early school age was associated with increasing probability of allocation to above average trajectory. Importantly, our results provide evidence of the association of diminished lung function trajectories with objective echocardiographic markers of the propensity to cardiovascular diseases (including heart failure and stroke). Our results add weight to the emerging concept in the field of assessing lung function in childhood (eg, at school) as a marker of subsequent risk of respiratory, cardiovascular and metabolic diseases.^4^

LVMI and cIMT are indicators of future cardiovascular disease risk. For example, cIMT has been extensively validated as a predictor of cardiovascular disease-risk in adults, and LVMI is a measure that independently predicts adverse cardiovascular events and premature death.^13 16^ We observed a relationship between decreasing lung function trajectory and an increase in both markers. Coronary Artery Risk Development in Young Adults (CARDIA) study was the first to investigate the interplay between early adulthood lung function and late cardiac changes demonstrated on an echocardiogram.^23^ A decline in FEV_1_/FVC was associated with decreased left heart chamber size and lower cardiac output, whereas a decline in FVC with a preserved FEV_1_/FVC (a precursor to a restrictive pathology) was associated with left heart hypertrophy, increased cardiac output, and diastolic dysfunction, irrespective of race, sex, age, height, cigarette smoking, diabetes or BMI.^23^ Although FEV_1_ and FVC are highly correlated, the fact that the pattern of airway pathology in young adulthood seems to differently influence future cardiovascular phenotypes could suggest an underlying mechanism which is independent of a systemic inflammatory response. However, while CARDIA aimed to identify factors in young adulthood that contribute to the development of cardiovascular disease, our studies followed participants from the antenatal period, allowing for more precise assessment of early-life risk factors, and the association with childhood lung function patterns.

The association between lung and cardiac disease has been long established in patients with COPD.^24^ There is a reduction in both cardiac chamber size and the left atrial and ventricular filling in those with severe COPD, with the degree of hyperinflation showing the strongest correlation with heart size. The mechanism behind this is unknown, but few studies have postulated that lung hyperinflation increases intrathoracic pressure, which decreases venous return,^24^ or increases LV wall stress leading to increased LV stroke work and eventual increased LV mass and LV remodelling.^25^ Indeed, the Multi-Ethnic Study of Atherosclerosis study found that an increase in LV mass-to-volume ratio as well as end-diastolic volume was associated with an increase in cardiovascular events, which is consistent with a load effect that induces chamber remodelling and hypertrophy.^26^ Other studies discussed a possible role of haemodynamic effects of hypoxia and vascular remodelling leading to pulmonary hypertension with subsequent effect on RV and LV interdependence as a cause of altered cardiac chamber size.^27^ Using CT scans, findings suggestive of ‘early emphysema’ in an otherwise healthy population were associated with lower LV end-diastolic volume, stroke volume and cardiac output, implying that the interaction between heart and lung seen in advanced disease initiates earlier in life and at subclinical levels of disease,^28^ likely prior to any of the proposed mechanisms in those with severe lung disease like COPD. This is supported by our findings. However, the above data come from cross-sectional studies in older populations, and our results take this one step further by identifying that lung function patterns starting in school age predict cardiac effect in adulthood.

Other major risk factors for cardiovascular disease include dyslipidaemia (defined as either high triglycerides, high low-density lipoprotein or low HDL). Of note, such metabolic abnormalities have also been associated with asthma^29–31^ and airway obstruction in children^32^.

Several studies have shown that poorer lung function in early adulthood is associated with stroke and hypertension in later adulthood. Lung function in young adulthood in CARDIA was independently associated with cardiovascular and cerebrovascular events into middle age,^33^ and within-individual change in lung function (including low normal and deterioration from peak health) was independently associated with a greater incidence of hypertension and blood pressure variability.^34^

In our study, low birth weight and maternal asthma were associated with persistently low FEV_1_/FVC trajectory. Similarly, we have previously demonstrated that low birth weight identified children with a persistently low FEV_1_ trajectory,^35^ as well as the restrictive phenotype.^6^ Both the IOW^36^ and the Pelotas cohorts^11^ showed that low birth weight was associated with low FEV_1_ and FEV_1_/FVC trajectories. Exact pathophysiological mechanisms have not been ascertained, but it has been suggested that adverse early life risk factors such as maternal smoking,^37^ poor maternal nutrition,^38^ restricted intrauterine growth^39^ and gestational age^40^ could contribute to this. This adds to the evidence that low birth weight acts as a proxy for adult health and that it is associated with chronic disease including coronary artery disease and hypertension.

Our study has several limitations. One limitation which is common to most analyses of longitudinal data which involve multiple follow-up measurements over a long period of time is missing values due to drop-out. Both analyses (complete dataset and at least two spirometry measures) gave consistent optimal goodness-of-fit using the BIC, and the child class assignments were stable across the two analyses. This suggests that the missing-at-random assumption was plausible, given that if children with missing datapoints were not missing at random, we would have observed a higher mismatch between classes.

A further limitation of our study is the heterogeneity between cohorts (minor differences in data collection ages, wording of questions, etc). However, the lung function trajectories were remarkably consistent across the cohorts. Another limitation is that missing data limits assessment of risk factors. Given a relatively small sample size in the persistently low trajectory, we may not have enough power to detect clinically important effects and may lack precision. Strengths of our study include the longitudinal nature of the data coming from multiple sources and covering different age ranges and screening intervals, the long duration of the follow-up (up to 26 years of age) and similar methodology applied for determining participants’ health status.

Our study highlights the importance of lung growth in general as the associate of adverse respiratory, cardiovascular and metabolic outcomes, and the importance of early life factors, particularly deprivation. Childhood lung function trajectories may serve as important predictors in the development of not only future lung disease, but also the interplay and multimorbidity of lung, cardiovascular and metabolic diseases. Our findings draw attention to the potential importance of measuring lung function from early school age as a marker of future risk, since early lung function optimisation to alter trajectories may help in preventing adverse health outcomes in adulthood. However, being able to identify a potential problem does not automatically extend to actionable interventions to address it. The question remains as to whether the age at which the majority of children in the community can perform reliable forced expiratory manoeuvres (usually around age 6 years) is already too late to intervene to improve lung growth.^41^ However, we have previously shown in ALSPAC that catch-up growth in FEV_1_ and FVC is possible around puberty, and that later onset and higher velocity of pubertal growth are associated with higher maximally attained lung function at age 24 years.^42^ Given the global trends towards earlier puberty^43^ and a significant relationship between the early onset of puberty with child’s obesity^44^ and maternal obesity and gestational weight gain,^45^ a combination of intervention tackling childhood obesity to protect current generations, and obesity in pregnancy to protect future generations (particularly among women with impaired lung function),^41^ may have substantial impact on overall health. This should be coupled with measures to minimise exposure to tobacco smoke, air pollution and other adverse environmental exposures. Among children with recurrent wheeze, every effort should be made to reduce the number of severe exacerbations. However, all these measures must be paralleled with societal efforts to reduce inequalities and social deprivation if the nation’s health is to be improved.

## Data Availability

Data are available upon reasonable request. The informed consent obtained from all included participants does not allow the data to be made freely available through any third party maintained public repository. However, data used for this submission can be made available on request to the corresponding cohort executive. The ALSPAC website provides information on how to request and access its data (http://www.bristol.ac.uk/alspac/researchers/access/). For queries regarding access of data from MAAS, IoW, SEATON or Ashford contact Philip Couch philip.couch@manchester.ac.uk).
