# Supplementary material for: Lung function trajectories from school age to adulthood and their relationship with markers of cardiovascular disease risk
Source: Thorax. 2024 May 2;79(8):770–7. doi: 10.1136/thorax-2023-220485 (PMC11287578; doi:10.1136/thorax-2023-220485)
Supplement: Supplementary data [file thorax-2023-220485supp001.pdf]

Lung function trajectories from school age to adulthood and their relationship with markers of cardiovascular disease risk

Raquel Granell, Sadia Haider, Matea Deliu, Anhar Ullah, Osama Mahmoud, Sara Fontanella, Lesley Lowe, Angela Simpson, James Dodd, Syed Hasan Arshad, Clare S Murray, Graham Roberts, Alun D Hughes, Chloe Park, John W Holloway, Adnan Custovic

Online Data Supplement

Granell R, *et al. Thorax* 2024; 79:770–777. doi: 10.1136/thorax-2023-220485

SUPPLEMENTARY INTRODUCTION

Table S1. *Summary of existing studies to derive lung function trajectories in childhood*

**Name of study**	MAAS[1]	ALSPAC[1]	PIAF[1]	TCRS[2]	IOW[3]	CAMP[4]	RAINE[5]	PELOTAS[6]	TAHS[7]
**Year of Publication**	2018	2018	2018	2016	2019	2016	2022	2020	2018
**Locations**	U.K.	U.K.	Australia	Arizona	UK	USA/Canada	Australia	Brazil	Tasmania/ Australia
**No. of subjects**	1046	1390	196 (spirometry on at least one occasion)<br>253 (infant lung function)	599 participants<br>with 2,142 observations	981 have LF at age 10, 839 have LF at age 18 and 547 individuals have LF at age 26	684	1512	2917 individuals who had measurements in all follow-up visits	2438
**Time points**	5, 8, 11, and 16 years	8, 15, and<br>24 years	6, 12, and 18 years &<br>1, 6, and 12 months	11, 16, 22, 26, and 32 years	10,18 and 26 years	23 - 30 years	6 – 22 years	15,18 and 22 years	7, 13, 18, 45, 87 50 and 53 years
**Spirometry Measures**	Forced expiratory volume in one second (FEV_1) and Forced vital capacity	Forced expiratory volume in one second (FEV_1) and Forced vital capacity	FEV_1 as % predicted at 6, 12, and 18 years.<br>Rapid thoracoabdo	Ratio of FEV_1 to FVC (FEV_1/FVC)	FVC, FEV_1, FEV_1 and FVC ratio and	Pre-BD FEV_1	FEV_1, FVC, FEV_1/FVC	FEV_1, FVC and FEV_1 and FVC ratio	Pre- and post-BD spirometry

	(FVC) were recorded and the data expressed as FEV_1 % predicted and FEV_1/FVC ratio.	(FVC) were recorded and the data expressed as FEV_1 % predicted and FEV_1/FVC ratio.	minal compression		FEF25-75					
			test to determine the maximal flow at functional residual capacity (V'maxFRC) at 1, 6, and 12 months.							
**Type of Spirometry Measures**	FEV1 as % predicted at each age (*2)	FEV1 as % predicted at each age (*2)	(1) Mean % predicted FEV_1 (95% CI) over time by FEV_1 trajectory (196 children).	Percent of predicted FEV_1 values.	Raw (Male and Female)	Pre-BD FEV_1	Forced expiratory volume in 1 s (FEV_1), forced vital capacity (FVC) and FEV_1/FVC (z-scores)	Z-scores	Pre-BD FEV_1 z-scores at six time points
			(2) Mean % predicted V'maxFRC (95% CI) over time by V'maxFRC trajectory (253 children).	Ratio of FEV_1 to FVC (FEV1/FVC), adjusting for sex.					

			(3) Membership of FEV_1 trajectory (from 6 to 18 years of age) in relation to the V'maxFRC trajectories (from 1 to 12 months of age), evaluated in 196 children.	V9maxFRC was measured at age 6 years.					
**Number of trajectories**	4	4	(1) 3;<br>(2) 3;<br>(3) 2	2	2 (FVC, FEV1, ratio) and 3 (FEF)	4	4	3	6
**Derived trajectories**	Persistently high, n=46; 4%,	Persistently high, n=52; 4%,	(1) Persistently high, n=24; 12%,	(1) Low: n = 56; 9.3%	Male FEV1:	Normal growth, n=70; 25%	FEV_1 and FVC trajectories<br>Very Low (n=51 3%, FEV_1, n=61 4% FVC)	Low: 15.5%,	(1) Early below average accelerated decline: 4%, n=97,
	Normal n=468; 45%,	Normal n=632; 45%,	Normal n=133; 68%,	(2) Normal: 543;	Low: 57.2%,	Normal growth, early decline, n=178; 26%	Low (n=746 49%, FEV_1, n=855 56% FVC)	Average: 59.6%,	(2) Persistently low: 5·6%, n=136,

	Below average n=496; 47%,	Below average n=613; 44%,	Below average n=39; 20%.		High: 42.8%.	Reduced growth, n=160; 23%	Average (n=611 40%, FEV_1, n=457 30% FVC)	High: 24.9%.	(3) Below average: 31·6%, n=772,
	Persistently low n=36; 3%.	Persistently low n=93; 7%.	(2) Above average n=62; 25%,		Female FEV1:	Reduced growth, early decline, n=176; 26%	Above Average (n=104 7%, FEV_1, n=139 9% FVC)		(4) Above average: 12·1%, n=293,
			Below average n=191; 75%.		Low: 47.9%,		FEV_1/FVC trajectories		(5) Early low, accelerated growth, normal decline: 8%, n=196,
							Very Low (n=131, 9%)		
			(3) V´max FRC: above average 25%		High: 52.1%.		Low Average (n=198, 13%)		(6) Average: 38·7%, n=944
							Average-Low (n=529, 35%)		
			V´max FRC: below average 75%				Average (n=654, 43%)		

**Methods used**	Two-level random intercept and random coefficients regression models	Two-level random intercept and random coefficients regression models	Two-level random intercept and random coefficients regression models	Latent class linear mixed effects model with subject specific random effects for slope and intercept and a fixed effect for sex.	Finite mixture models	Subjective expert classificatio n using NHANES III, Kaplan-Meier	Group based trajectory modeling	Group based trajectory modeling	Group-based trajectory modelling

SUPPLEMENTARY METHODS

The Avon Longitudinal Study of Parents and Children (ALSPAC)

ALSPAC is a birth cohort study established in 1991 in Avon, UK[8-10]. Pregnant women with expected dates of delivery 1st April 1991 to 31st December 1992 were invited to take part in the study. The initial number of pregnancies enrolled is 14,541. Of these initial pregnancies, there was a total of 14,676 foetuses, resulting in 14,062 live births and 13,988 children who were alive at 1 year of age.

When the oldest children were approximately 7 years of age, an attempt was made to bolster the study with eligible cases who had failed to join originally. As a result, when considering variables collected from the age of seven onwards (and potentially abstracted from obstetric notes) there are data available for more than the 14,541 pregnancies mentioned above. The number of new pregnancies not in the initial sample (known as Phase I enrolment) that are currently represented on the built files and reflecting enrolment status at the age of 24 is 913 (456, 262 and 195 recruited during Phases II, III and IV respectively), resulting in an additional 913 children being enrolled. The phases of enrolment are described in more detail in the cohort profile paper and its update. The total sample size for analyses using any data collected after the age of seven is therefore 15,454 pregnancies, resulting in 15,589 foetuses. Of these 14,901 were alive at 1 year of age.

Study data were collected and managed using REDCap Research Electronic Data Capture) electronic data capture tools[11] hosted at University of Bristol.

Ethical approval for the study was obtained from the ALSPAC Ethics and Law Committee and the Local Research Ethics Committees. Informed consent for the use of data collected via questionnaires and clinics was obtained from participants following the recommendations of the ALSPAC Ethics and Law Committee at the time. The study website contains details of available data through a fully searchable data dictionary and variable search tool: http://www.bristol.ac.uk/alspac/researchers/our-data/

We are extremely grateful to all the families who took part in this study, the midwives for their help in recruiting them, and the whole ALSPAC team, which includes interviewers, computer and laboratory technicians, clerical workers, research scientists, volunteers, managers, receptionists and nurses.

The Manchester Asthma and Allergy Study (MAAS)

MAAS is an unselected birth cohort study established in 1995 in Manchester, UK[12]. It consists of a mixed urban-rural population within 50 square miles of South Manchester and Cheshire, located within the maternity catchment area of Wythenshawe and Stepping Hill Hospitals. All pregnant women were screened for eligibility at antenatal visits (8-10th week of pregnancy). Of the 1499 couples who met the inclusion criteria (≤10 weeks of pregnancy, maternal age ≥18 years, and questionnaire and skin prick data test available for both parents), 288 declined to take part in the study and 27 were lost to follow-up between recruitment and the birth of a child. A total of 1184 children were born into the study between February 1996 and April 1998. They were followed prospectively for 20 years to date and attended follow-up clinics for assessments, which included lung function measurements, skin prick testing, biological samples (serum, plasma and urine), and questionnaire data collection. The study was approved by the North West – Greater Manchester East Research Ethics Committee.

We capitalized on a unique feature of the health care system in the UK in that, General practitioners (GPs) maintain primary care records of all health care encounters of their patients, including hospital admission, and outpatient appointments. A trained paediatrician extracted and transcribed data from GP-held medical records including AD diagnosis and prescriptions for topical treatments. Timing, type of visit, symptoms, indication and prescriptions for each encounter were noted. A total of 987 participants

provided informed consent for medical data collection. We reviewed 925 study participants due to GPs' lack of response for data collection or participants moving away. Nine of these were partially accessed due to missing paper or electronic records and were excluded.

Data on lower respiratory tract infections (LRTI), hospital admissions, bronchiolitis, and RSV-positive bronchiolitis were extracted from electronic and paper-based primary care medical records, including emergency department admissions, and hospital admissions. Age in days at the time of each event was documented63. This data was available from birth to age 8 years.

Atopic sensitization was ascertained by skin prick testing (SPT) at age 8, 11, 16, and 20 years and measurement of sIgE at each clinical follow-up to a mix of common inhalant and food allergens by ImmunoCAPTM (Phadia, Uppsala, Sweden).

We thank study participants and their parents for their continued support and enthusiasm, and greatly appreciate the commitment they have given to the project. We also acknowledge the hard work and dedication of the study teams (post-doctoral scientists, physiologists, research fellows, nurses, technicians, and clerical staff).

The Isle of Wight (IOW) cohort

IOW is an unselected birth cohort study established in 1989 on the Isle of Wight, UK[13-15]. After the exclusion of adoptions, perinatal deaths, and refusal for follow-up, written informed consent was obtained from parents to enrol 1,456 newborns (of 1536 born between 1st January 1989 and 28th February 1990). Follow-up-up assessments were conducted to 26 years of age to prospectively study the development of asthma and allergic diseases. At each follow-up, validated questionnaires were completed by the parents. Additionally, the Skin Prick Test (SPT) was performed on 980, 1036 and 853 participants at 4, 10 and 18 years of age to check allergic reactions to common allergens. Ethics approvals were obtained from the Isle of Wight Local Research Ethics Committee (now named the National Research Ethics Service, NRES Committee South Central – Southampton B) at recruitment and for the subsequent follow-ups.

The IOW research team are grateful to all the participants and their families for their support over the years and also to the many fellow researchers who have contributed to the cohort's follow up.

Spirometry

ALSPAC

Spirometry tests were were conducted at $8^{1/2}$, 15 and 24 years according to American Thoracic Society/European Respiratory Society guidelines[16,17] using a Vitalograph pneumotachograph system with animated incentive software (Spirotrac, Vitaograph, UK) in a dedicated research clinic by trained technicians. Calibration checks were performed with a standard 3L calibration syringe according to the manufacturer's instructions at the start of each half-day clinic session. Subjects were seated with a nose clip in place and were asked to inhale to total lung capacity (TLC), then instructed to perform a forced expiration, through a mouthpiece, to residual volume (RV). The test was repeated at intervals of 30 seconds until 3 technically acceptable traces were obtained from a maximum of eight attempts. Forced expiratory volume in one second (FEV_1) and Forced vital capacity (FVC) were recorded and the data expressed as FEV_1 % predicted and FEV_1/FVC ratio.

MAAS

Spirometry was performed at ages 8, 11, 16 and 20 years according to American Thoracic Society/European Respiratory Society guidelines[16,17] using a Lilly pneumotachograph system with animated incentive software (Jaeger, Germany). For home visits, we used a flow turbine spirometer

(Micro Medical, UK). Subjects were asked to inhale to total lung capacity (TLC), then instructed to perform a forced expiration, through a mouthpiece, to residual volume (RV). The test was repeated at intervals of 30 seconds until 3 technically acceptable traces were obtained. Forced expiratory volume in one second (FEV_1) and Forced vital capacity (FVC) were recorded and the data expressed as FEV_1 % predicted and FEV_1/FVC ratio. Short-acting β2-agonists were withheld for at least four, and long-acting for at least 24 hours prior to testing. Participants were symptom-free at the time of assessment.

IOW

Pre-bronchodilator lung function tests were conducted at 10, 18, and 26 years of age. Forced vital capacity (FVC), forced expiratory volume in 1 second (FEV1) were measured using a Koko Spirometer and software with a portable desktop device (both PDS Instrumentation, Louisville, KY, USA). Spirometry was performed and evaluated according to the American Thoracic Society (ATS) criteria. The children or adults, respectively, were required to be free of respiratory infection for 2 weeks and not to be taking any oral steroids and were advised to abstain from any β-agonist medication for 6 h and from caffeine intake for at least 4 h.

Assessment of cardiovascular risk in ALSPAC

Participants fasted for 6 h before the clinic, with the exception of those with a diagnosis of diabetes or a condition that would not allow fasting. Ultrasound scans of the left and right common carotid arteries were performed using a CardioHealth Panasonic system with a 13–5 MHz linear array broadband transducer according to a standardised protocol to measure cIMT. Participants lay on a couch with their arms by their side, while a trained researcher performed the ultrasound test on both sides of their neck. Right and left carotid intima-media thickness measurements were taken to be the average of 3 end-diastolic measurements of the far-wall of the common carotid artery over a length of 5–10 mm, and 10 mm adjoining the bifurcation. The mean of both right and left cIMT measures was calculated.

Right and left carotid intima-media thickness measurements were taken to be the average of 3 end-diastolic measurements of the far-wall of the common carotid artery over a length of 5–10 mm, and 10 mm adjoining the bifurcation. The mean of both right and left cIMT measures was calculated.

Echocardiography was performed by two experienced echocardiographers using a Philips EPIQ 7G Ultrasound System equipped with a X5-1 transducer and methods and calculation were performed in accordance with American Society of Echocardiography guidelines; these techniques are described elsewhere[18]. PWV was measured using a Vicorder device (Skidmore Medical, Bristol, UK) which has been validated in previous studies in adolescents [19]. Three pulse wave velocity measurements were taken with an interval of 1 minute between measurements, acceptable PWV measurements were within ≤ 0.5 m/s of each other. Results were averaged to give a measurement of arterial stiffness. Resting blood pressure was measured with the subject in a sitting position using an Omron 705-IT machine using an appropriately sized cuff [18] as the average of the final two of three consecutive measurements.

Resting blood pressure (BP) was measured in a sitting position as the average of the final two of three consecutive measurements.

Definitions of variables (demographic, exposures and outcomes)

Postal questionnaires were used in ALSPAC, while interviewer-administered questionnaires were employed in MAAS and IOW available on multiple occasions from infancy to adolescence.

Wheezing reports were available at 14 time points in ALSPAC over 16.5 years, 7 in MAAS over 20 years, and 6 in IOW over 26 years.

Parental history of asthma, eczema and hay fever were assessed by questionnaires and were defined based on the responses given to the question "have you (and/or your partner) ever had asthma/eczema/hay fever".

Maternal and paternal smoking were defined based on the response given to the question "do you (or does your partner) smoke", administered during pregnancy or 1st year of study child.

Low birth weight was defined as birth weight less than 2500 g based on NHS birth records.

Current wheeze: Current wheeze was defined as a positive response to either the question "Has your child had wheezing in the last 12 months" or "Has your child had wheezing with whistling in the last 12 months" in ALSPAC, and in the other three cohorts to the question "Has your child had wheezing or whistling in the chest in the last 12 months?".

Current asthma[20]*:* Presence of any two of the following three features: 1) Current wheeze; 2) Current use of asthma medication; 3) Physician-diagnosed asthma ever.

BMI (kg/m^2): weight and height were measured at annual clinic visits

Skin prick test (SPT): The atopic status of the children was determined (at an annual clinic when the children were 7–8 years of age in ALSPAC[21], at ages 4-5, and 7-8 years in MAAS and IOW) by skin prick test responses to a panel of up to 12 common allergens including house dust mite, mixed grasses and cat. Sensitisation to one of these three allergens has been shown to identify 95% of all sensitised children in this population. A positive response was defined as a mean weal diameter of >3 mm (>2 mm for ALSPAC) with an absent response to negative control solution, and atopy was defined as a positive response to one or more of house dust mite, cat or grass pollen.

<u>MAAS transcribed healthcare records[22]</u>

LRTI hospitalisation: Lower respiratory tract infection hospital admission by 3rd year of life.

RSV positive bronchiolitis: ever RSV positive bronchiolitis in the first 8 years of life.

Asthma/wheeze hospital admission: asthma or wheee hospital admission by 3rd year of life.

Data-driven phenotype allocation

Cluster allocation of our study participants into latent wheeze phenotypes has previously been performed using machine learning methods for latent variable analysis.

Spell-based wheeze phenotypes[23]*:* Based on prospectively collected current wheeze data from five pooled birth cohorts birth to age 16 years , children were assigned as:

1. Never wheezing: no or low prevalence of wheeze throughout observation period.
2. Early-transient wheeze: high prevalence of wheeze during infancy, with decrease to mid-childhood.
3. Intermittent wheeze: wheeze from infancy to adolsence interspersed with periods of no wheeze.
4. Late-onset wheezing: low prevalence until mid-childhod age, increasing rapidly to a peak prevalence in adolescence.
5. Persistent wheeze*:* high prevalence of wheeze throughout.

Statistical Analysis

<u>*Latent profile modeling*[1,24] *to derive trajectory classes based on the development of FEV_1/FVC over time*</u>

We used two-level random intercept regression models to assign children to their most likely trajectory profile; we assumed that each child belonged to one of a set of *k* latent profiles, the number or size of

which were unknown a priori. The models were compared for goodness-of-fit using the Bayesian Information Criterion (BIC). For each child, the posterior probability of belonging to each of the classes was estimated from the model, and children were classified to each trajectory profile based on their maximum posterior probability. Latent profile modelling was undertaken using the *gllamm* (generalised linear latent and mixed models) package implemented in Stata 15 (StataCorp, College Station, TX, USA).

Associations between lung function trajectories and early−life risk factors

The following potential predictors, as evidenced in the literature, were considered in analyses with the derived lung function trajectories: gender, low birthweight, maternal smoking during pregnancy, parental history of asthma, cat/dog ownership in first year of life, current wheeze and current asthma reports from birth to age 8 years, LRTI/asthma/wheeze hospitalizations by age 3 and RSV positive bronchiolitis (MAAS only), BMI and allergic sensitization from early life to age 8 years.

Associations between lung function trajectories and markers of cardiovascular and metabolic disease risk

We used linear regression models to assess the associations between lung function trajectories between 8 and 24 years and markers of cardiovascular and metabolic disease risk at 24 years, before and after adjustment by gender, maternal lower education level (Educated to the General Certificate of Education level (school-leaving certificate) or lower) and child's BMI at 7 years. We report both individual trajectory effects using average as the reference category and per-trajectory effects treating lung function trajectories as a continuous variable (1. Above average; 2. Average; 3. Below average; and 4. Persistently low).

All cardiovascular outcomes were normally distributed except for Triglycerides, which was log-transformed.

All analyses were carried out using Stata 16/17 (StataCorp, College Station, Tex).

Granell R, *et al. Thorax* 2024; 79:770–777. doi: 10.1136/thorax-2023-220485

SUPPLEMENTARY RESULTS

Table S2. *Characteristics of the study populations: MASS, IOW, and ALSPAC*

	Whole population	Included: Children with data on lung function at 2-3 points	Excluded: Children with data on lung function at <2 time points	P-value included vs excluded
**MAAS**	**N=1184**	**N=801**	**N=383**	
Parental characteristics				
Maternal age (mean/SD)	30.4 (4.78)	30.8 (4.58)	29.58 (5.12)	0.0001
Maternal asthma	19.9% (235/1184)	20.0% (160/801)	19.6% (75/383)	0.874
Paternal asthma	13.8% (163/1182)	13.6% (109/800)	14.1% (54/382)	0.812
Perinatal characteristics				
Male gender	54.2% (642/1184)	52.4% (420/801)	58.0% (222/383)	0.074
Low birth weight (≤2500 gr)	3.0% (34/1136)	3.1% (24/773)	2.6% (10/363)	0.747
Environmental characteristics				
Breastfeeding	70.0% (780/1115)	73.8% (569/771)	61.3% (211/344)	P<0.0001
Maternal smoking during pregnancy	14.8% (174/1177)	12.2% (97/798)	20.3% (77/379)	P<0.0001
Paternal smoking during pregnancy	29.4% (347/1181)	28.0% (224/799)	32.2% (123/382)	0.142
Presence of cat (recruitment)	20.7% (240/1160)	20.5% (161/787)	21.2% (79/373)	0.777
Presence of dog (recruitment)	17.3% (201/1160)	15.3% (120/787)	21.7% (81/373)	0.007
**Isle of Wight (IoW)**	**N=1536**	**N=809**	**N=727**	
Parental characteristics				
Maternal age (mean/SD)	26.8 (5.54)	27.1 (5.27)	26.39 (5.43)	0.009
Maternal asthma	10.9% (165/1517)	10.5% (84/802)	11.3% (81/715)	0.593
Paternal asthma	9.9% (149/1504)	10.2% (81/798)	9.6% (68/706)	0.737
Perinatal characteristics				
Male gender	51.2% (786/1536)	46.6% (377/809)	56.3% (409/727)	P<0.0001
Low birth weight (≤2500 gr)	4.1% (62/1501)	3.8% (30/791)	4.5% (32/710)	0.487
Environmental characteristics				
Breastfeeding	77.% (1047/1346)	82.8% (625/755)	71.4% (422/591)	P<0.0001
Maternal smoking during pregnancy	26.0% (393/1509)	19.9% (159/798)	32.9% (234/711)	P<0.0001
Paternal smoking during pregnancy	40.5% (608/1503)	34.4% (275/802)	47.3% (333/704)	P<0.0001

Granell R, et al. Thorax 2024; 79:770–777. doi: 10.1136/thorax-2023-220485

Table S2 continue

	Whole population	Included: Children with data on lung function at 2-3 points	Excluded: Children with data on lung function at <2 time points	P-value included vs excluded
Presence of cat (recruitment)	32.6% (494/1514)	33.8% (271/802)	31.3% (223/712)	0.306
Presence of dog (recruitment)	29.2% (442/1514)	28.3% (227/802)	30.2% (215/712)	0.419
**ALSPAC**	**N=15,645**	**N=4,767**	**N=10,878**	
Parental characteristics				
Maternal age (mean/SD)	28.0 (5.0), N=14049	29.4 (4.5), N=4559	27.3 (5.0), N=9490	P<0.0001
Maternal asthma ever	11.4% (1435/12590)	11.7% (521/4437)	11.2% (914/8153)	0.37
Paternal asthma ever	12.7% (1092/8568)	12.9% (436/3381)	12.6% (656/5187)	0.74
Perinatal characteristics				
Male gender	51.2% (7706/15061)	46.0% (2193/4767)	53.6% (5513/10294)	P<0.0001
Low birth weight (≤2500 gr)	5.7% (790/13867)	4.3% (194/4503)	6.4% (596/9364)	P<0.0001
Environmental characteristics				
Breastfeeding 1st year	75.7% (8573/11332)	84.4% (3655/4331)	70.2% (4918/7001)	P<0.0001
Maternal smoking 1st year	24.2% (2714/11219)	15.7% (676/4294)	29.4% (2038/6925)	P<0.0001
Paternal smoking 1st year	26.2% (2208/8434)	20.4% (694/3402)	30.1% (1514/5032)	P<0.0001
Presence of cat 1st year	32.7% (2525/7733)	34.9% (1073/3075)	31.2% (1452/4658)	0.0006
Presence of dog 1st year	25.1% (1747/6955)	22.9% (594/2596)	26.5% (1153/4359)	0.0009

Granell R, *et al. Thorax* 2024; 79:770–777. doi: 10.1136/thorax-2023-220485

Table S3. *Characteristics of latent class profiles of FEV1/FVC in three cohorts using children with data on 2 or 3 time points: model comparison of goodness-of-fit using Bayesian Information Criterion (BIC).*

**BIC (FEV1/FVC)**			
Cohort	**MAAS** (N=801)	**IOW** (N=809)	**ALSPAC** (N=4767)
Number of classes			
2	16417	12959	75239
3	16258	12843	74785
4	16203	12820	74692
5	16200	12820	74701
6	*	*	74720

Granell R, *et al. Thorax* 2024; 79:770–777. doi: 10.1136/thorax-2023-220485

Table S4. *Posterior probability of the class membership conditional on most likely class assignment for FEV1/FVC (Class 1: Below Average; Class 2: Average; Class 3: Persistently Low; Class 4: Above average)*

		MAAS Class Assignment				IOW Class Assignment				ALSPAC Class Assignment			
		1	*2*	*3*	*4*	*1*	*2*	*3*	*4*	*1*	*2*	*3*	*4*
Class Membership	*1*	*0.87*	*0.13*	*0.00*	*0.00*	*0.81*	*0.16*	*0.03*	*0.00*	*0.70*	*0.19*	*0.03*	*0.00*
	2	*0.05*	*0.82*	*0.00*	*0.13*	*0.07*	*0.77*	*0.00*	*0.16*	*0.09*	*0.73*	*0.00*	*0.20*
	3	*0.09*	*0.00*	*0.91*	*0.00*	*0.19*	*0.00*	*0.81*	*0.00*	*0.15*	*0.00*	*0.81*	*0.00*
	4	*0.00*	*0.16*	*0.00*	*0.84*	*0.00*	*0.13*	*0.00*	*0.87*	*0.00*	*0.17*	*0.00*	*0.84*

Table S5. *Confusion matrix showing similarity in classification when using children with FEV1/FVC on 2 or 3 time points, and classification based on information from children with data at all 3 time points.*

	Class assignment model based on children with FEV1/FVC data at 2 or 3 time points (ARI=0.63)											
	**MAAS (N=346 with data at 3 time points)**				**IOW (N=401 with data at 3 time points)**				**ALSPAC (N=2033 with data at 3 timepoints)**			
Class assignment model based on children with on FEV1/FVC data observed at all 3 time points	Peristently High	Average	Below Average	Persistently Low	Peristently High	Average	Below Average	Persistently Low	Peristently High	Average	Below Average	Persistently Low
Persistently High	105 (78.4%)	0	0	0	163 (100%)	0	0	0	827 (100%)	185 (22.2%)	0	0
Average	29 (21.6%)	140 (90.3%)	0	0	0	179 (100%)	0	0	0	648 (77.8%)	102 (32.0 %)	0
Below Average	0	15 (9.7%)	43 (84.3%)	0 (0%)	0	0	48 (100%)	0	0	0	217 (68.0%)	21 (38.9%)
Persistently Low	0	0	8 (15.7%)	6 (100%)	0	0	0	11 (100%)	0	0	0	33 (61.1 %)

Granell R, *et al. Thorax* 2024; 79:770–777. doi: 10.1136/thorax-2023-220485

***Table S6**. Sex, demographic and environmental characteristics of FEV_1/FVC trajectories: multinomial logistic regression analysis weighted by class membership probabilities; reference class is Average*

	Relative Risk Ratio (95%CI)								
	MAAS (Average n=379; 47.3%)			IOW (Average n=368; 45.5%)			ALSPAC (Average n=1816; 38.1%)		
	Above average (n=309; 38.6%)	Below average (n=100; 12.5%)	Persistently low (n=13; 1.6%)	Above average (n=320; 39.6%)	Below average (n=97; 12.0%)	Persistently low (n=24; 3.0%)	Above average (n=2355; 49.4%)	Below average (n=516; 10.8%)	Persistently low (n=80; 1.7%)
Male	0.54 (0.4, 0.74)	1.66 (1.04, 2.64)	9.35 (1.2, 72.65)	0.71 (0.52, 0.96)	1.60 (1.02, 2.53)	1.80 (0.77, 4.21)	0.64 (0.57, 0.73)	1.59 (1.31, 1.92)	2.10 (1.32, 3.33)
p value	<0.001	0.034	0.033	0.027	0.041	0.18	3.26E-12	1.87E-06	0.0016
Preterm (<37 weeks gestation)	NA	NA	NA	NA	NA	NA	0.73 (0.54, 0.98)	1.11 (0.74,1.67)	1.53 (0.67, 3.48)
p value							0.04	0.62	0.31
Low birth weight (<2500 g)	0.68 (0.28, 1.63)	0.53 (0.12, 2.37)	NA	1.49 (0.65, 3.46)	2.34 (0.83, 6.61)	1.51 (0.19, 12.3)	0.89 (0.65, 1.23)	1.40 (0.91, 2.15)	2.30 (1.05, 5.06)
p value	0.39	0.41	0.99	0.35	0.11	0.70	0.49	0.12	0.038
Maternal smoking (pregnancy or 1st year)	1.06 (0.67, 1.68)	1.13 (0.58, 2.19)	1.37 (0.29, 6.39)	1.02 (0.69, 1.5)	1.34 (0.79, 2.3)	2.16 (0.89, 5.26)	0.89 (0.74, 1.06)	1.30 (1.01, 1.67)	1.60 (0.93, 2.76)
p value	0.81	0.72	0.69	0.93	0.28	0.089	0.20	0.043	0.09
Paternal smoking (1st year)	0.74 (0.53, 1.04)	0.74 (0.45, 1.21)	0.98 (0.29, 3.27)	NA	NA	NA	1.00 (0.83, 1.20)	1.15 (0.88, 1.51)	1.50 (0.86,2.64)
p value	0.08	0.23	0.97				0.99	0.31	0.16
Paternal history asthma	1.06 (0.67, 1.68)	2.02 (1.14, 3.59)	0.63 (0.08, 4.98)	1.34 (0.8, 2.24)	1.71 (0.86, 3.43)	2.21 (0.71, 6.9)	0.87 (0.70, 1.08)	1.08 (0.78, 1.49)	1.01 (0.49, 2.09)
p value	0.81	0.017	0.66	0.27	0.13	0.17	0.22	0.64	0.99
Maternal history asthma	1.09 (0.75, 1.59)	1.11 (0.65, 1.92)	0.76 (0.17, 3.51)	0.93 (0.56, 1.54)	1.51 (0.78, 2.93)	1.27 (0.36, 4.45)	0.90 (0.74, 1.10)	1.14 (0.86, 1.53)	1.88 (1.07, 3.30)
p value	0.64	0.70	0.73	0.77	0.22	0.71	0.29	0.37	0.027
Cat ownership (pregnancy or year 1)	1.02 (0.7, 1.47)	0.81 (0.45, 1.45)	0.31 (0.04, 2.45)	0.92 (0.67, 1.27)	1.3 (0.82, 2.06)	2.38 (1.04, 5.48)	0.97 (0.85, 1.10)	1.05 (0.86, 1.28)	1.45 (0.93, 2.27)
p value	0.93	0.48	0.27	0.61	0.27	0.041	0.63	0.65	0.10
Dog ownership (pregnancy)	1.17 (0.76, 1.79)	1.66 (0.93, 2.94)	0.52 (0.07, 4.12)	0.74 (0.53, 1.03)	0.54 (0.31, 0.92)	0.54 (0.20, 1.49)	0.95 (0.83, 1.08)	1.02 (0.83, 1.24)	0.92 (0.58, 1.46)
p value	0.47	0.085	0.54	0.071	0.024	0.24	0.43	0.88	0.72

Granell R, *et al. Thorax* 2024; 79:770–777. doi: 10.1136/thorax-2023-220485

Table S6 continue

	Relative Risk Ratio (95%CI)								
	**MAAS (Average n=379; 47.3%)**			**IOW (Average n=368; 45.5%)**			**ALSPAC (Average n=1816; 38.1%)**		
	**Above average (n=309; 38.6%)**	**Below average (n=100; 12.5%)**	**Persistently low (n=13; 1.6%)**	**Above average (n=320; 39.6%)**	**Below average (n=97; 12.0%)**	**Persistently low (n=24; 3.0%)**	**Above average (n=2355; 49.4%)**	**Below average (n=516; 10.8%)**	**Persistently low (n=80; 1.7%)**
BMI (kg/m2, age 5)	0.96 (0.87, 1.07)	1.23 (1.09, 1.4)	0.99 (0.68, 1.44)	0.91 (0.81, 1.03)	1.03 (0.87, 1.23)	1.07 (0.79, 1.44)	NA	NA	NA
p value	0.47	0.001	0.95	0.13	0.71	0.66			
BMI (kg/m2, age 7-8)	0.95 (0.89, 1.02)	1.09 (1.01, 1.19)	1.02 (0.81, 1.28)	0.96 (0.91, 1.01)	1.02 (0.94, 1.09)	0.96 (0.83, 1.12)	0.92 (0.89, 0.95)	1.02 (0.98, 1.07)	1.08 (0.98, 1.19)
p value	0.03	0.16	0.89	0.14	0.68	0.60	1.11E-06	0.32	0.11

Granell R, *et al. Thorax* 2024; 79:770–777. doi: 10.1136/thorax-2023-220485

Table S7. *Early-life characteristics of FEV_1/FVC trajectories: multinomial logistic regression analysis weighted by class membership probabilities; reference class is Average*

	**Unadjusted Relative Risk Ratio (95%CI)**								
	**MAAS (Average n=379; 47.3%)**			**IOW (Average n=368; 45.5%)**			**ALSPAC (Average n=1816; 38.1%)**		
	**Above average (n=309; 38.6%)**	**Below average (n=100; 12.5%)**	**Persistently low (n=13 1.6%)**	**Above average (n=320; 39.6%)**	**Below average (n=97; 12.0%)**	**Persistently low (n=24; 3.0%)**	**Above average (n=2355; 49.4%)**	**Below average (n=516; 10.8%)**	**Persistently low (n=80; 1.7%)**
**WHEEZE AND ASTHMA**									
Current wheeze (Age 4-5)	0.84 (0.57, 1.24)	2.61 (1.62, 4.21)	3.88 (1.22, 12.37)	0.97 (0.61, 1.54)	2.89 (1.66, 5.05)	3.6 (1.49, 8.72)	0.78 (0.64, 0.95)	2.04 (1.59, 2.61)	3.26 (2.01, 5.30)
p value	0.373	0	0.022	0.902	<0.001	0.005	0.013	p<0.001	p<0.001
Current wheeze (Age 7-8)*	0.74 (0.48, 1.15)	3.57 (2.19, 5.8)	2.26 (0.67, 7.56)	0.7 (0.47, 1.05)	2.61 (1.6, 4.24)	2.97 (1.27, 6.96)	0.63 (0.51, 0.78)	1.81 (1.41, 2.34)	3.95 (2.41, 6.47)
p value	0.182	<0.001	0.187	0.088	<0.001	0.012	p<0.001	p<0.001	p<0.001
Current Asthma (Age 4-5)	0.64 (0.42, 0.95)	2.36 (1.46, 3.81)	3.76 (1.18, 11.97)	0.82 (0.53, 1.3)	2.76 (1.61, 4.73)	3.31 (1.37, 7.98)	NA	NA	NA
p value	0.029	<0.001	0.025	0.403	<0.001	0.008	NA	NA	NA
Current Asthma (Age 7-8)*	0.75 (0.48, 1.15)	2.72 (1.65, 4.5)	3.25 (1.03, 10.26)	0.68 (0.45, 1.03)	2.64 (1.62, 4.3)	2.71 (1.13, 6.51)	0.71 (0.57, 0.87)	1.92 (1.47, 2.50)	3.62 (2.14, 6.11)
p value	0.187	0	0.045	0.068	<0.001	0.026	0.001	p<0.001	p<0.001
LRTI hospitalisation y age 3	0.48 (0.29, 0.8)	1.97 (1.11, 3.47)	3.41 (1.07, 10.82)	NA	NA	NA	NA	NA	NA
p value	0.005	0.02	0.037	NA	NA	NA	NA	NA	NA
Asthma/wheeze hospitalisation by age 3	0.42 (0.18, 0.96)	3.47 (1.73, 6.97)	4.36 (1.12, 17.01)	NA	NA	NA	NA	NA	NA
p value	0.039	<0.001	0.034	NA	NA	NA	NA	NA	NA
Ever RSV positive	0.8 (0.28, 2.26)	3.41 (1.23, 9.44)	6.73 (1.3, 34.88)	NA	NA	NA	NA	NA	NA
p value	0.668	0.018	0.023	NA	NA	NA	NA	NA	NA

Granell R, *et al. Thorax* 2024; 79:770–777. doi: 10.1136/thorax-2023-220485

Table S7 continue

	**MAAS (Average n=379; 47.3%)**			**IOW (Average n=368; 45.5%)**			**ALSPAC (Average n=1816; 38.1%)**		
	**Above average (n=309; 38.6%)**	**Below average (n=100; 12.5%)**	**Persistently low (n=13 1.6%)**	**Above average (n=320; 39.6%)**	**Below average (n=97; 12.0%)**	**Persistently low (n=24; 3.0%)**	**Above average (n=2355; 49.4%)**	**Below average (n=516; 10.8%)**	**Persistently low (n=80; 1.7%)**
**ALLERGIC SENSITIZATION**									
Sensitisation (SPT) (Age 4-5)	0.61 (0.42, 0.87)	1.23 (1.09, 1.4)	0.99 (0.68, 1.44)	0.75 (0.47, 1.2)	1.97 (1.11, 3.5)	3.84 (1.44, 10.21)	NA	NA	NA
p value	0.007	0.001	0.946	0.235	0.02	0.007	NA	NA	NA
Sensitisation (SPT) (Age 7-8)	0.6 (0.43, 0.84)	1.09 (1.01, 1.19)	1.02 (0.81, 1.28)	0.87 (0.61, 1.25)	1.72 (1.06, 2.79)	1.8 (0.76, 4.31)	0.93 (0.78, 1.10)	1.49 (1.17, 1.90)	2.41 (1.44, 4.03)
p value	0.003	0.055	0.082	0.46	0.027	0.184	0.39	1.18E-03	7.64E-04

* Year 10 for IOW; RSV Respiratory syncytial virus; SPT Skin Prick Test

Granell R, *et al. Thorax* 2024; 79:770–777. doi: 10.1136/thorax-2023-220485

Table S8. *Comparison of FEV_1/FVC trajectories and spell-based partition-around-medoids (PAM)phenotypes [23] (N and column %) using most likely class assignment*

WHEEZE PHENOTYPES-PAM joint	MAAS Peristently High	MAAS Average	MAAS Below Average	MAAS Peristently Low	MAAS Total	IOW Peristently High	IOW Average	IOW Below Average	IOW Peristently Low	IOW Total
Never wheeze	180	185	30	2	397	198	195	33	6	432
	58.3	48.8	30	15.4	49.6	61.9	53	34	25	53.4
Early-transient wheeze	73	94	20	4	191	45	50	15	3	113
	23.6	24.8	20	30.8	23.9	14.1	13.6	15.5	12.5	14
Intermittent wheeze	22	32	15	2	71	16	22	5	3	46
	7.1	8.4	15	15.4	8.9	5	6	5.2	12.5	5.7
Late onset wheeze	15	24	10	0	49	40	75	21	5	141
	4.9	6.3	10	0	6.1	12.5	20.4	21.7	20.8	17.4
Persistent wheeze	19	44	25	5	93	21	26	23	7	77
	6.2	11.6	25	38.5	11.6	6.6	7.1	23.7	29.2	9.5
Total	309	379	100	13	801	320	368	97	24	809
	100	100	100	100	100	100	100	100	100	100

MAAS: Chi-square test p<0.001

IOW: Chi-square test p<0.001

Table S8 continue

**WHEEZE PHENOTYPES-PAM joint**	**Peristently High**	**Average**	**Below Average**	**Peristently Low**	**Total**
			**ALSPAC**		
Never wheeze	370	310	96	16	792
	23.52	25.9	26.82	27.59	24.86
Early-transient wheeze	72	75	41	9	197
	4.58	6.27	11.45	15.52	6.18
Intermittent wheeze	78	71	25	7	181
	4.96	5.93	6.98	12.07	5.68
Late onset wheeze	971	646	138	11	1766
	61.73	53.97	38.55	18.97	55.43
Persistent wheeze	82	95	58	15	250
	5.21	7.94	16.2	25.86	7.85
Total	1573	1197	358	58	3186
	100	100	100	100	100

Chi-square test $p<0.001$

Granell R, *et al. Thorax* 2024; 79:770–777. doi: 10.1136/thorax-2023-220485

Table S9. *Associations between FEV1/FVC trajectories (8 to 24 years) and blood pressure at 20 years in 476-492 individuals in MAAS. Lung function trajectories treated as a) multinomial with Average as the reference class, and b) as continuous: 1. Above average (38.6%); 2. Average (47.3%); 3. Below average (12.5%); and 4. Persistently low (1.6%). Linear regression crude and adjusted analyses weighted by class membership probabilities.*

a) Lung function trajectories included as multinomial predictor with Average as the reference class

	Systolic blood pressure (mmHg)		Diastolic blood pressure (mmHg)	
	Mean difference p-value	95% CI	Mean difference p-value	95% CI
**Crude N=492**				
**Above Average**	-2.55<br>0.034	[-4.89,-0.20]	0.22<br>0.780	[-1.35,1.80]
**Average**	0 [ref]		0 [ref]	
**Below Average**	1.08<br>0.526	[-2.26,4.42]	0.64<br>0.574	[-1.60,2.88]
**Persistently Low**	5.05<br>0.209	[-2.83,12.94]	2.69<br>0.317	[-2.59,7.97]
**ADJUSTED by gender, BMI at 8 years and maternal smoking N=476**				
**Above Average**	-1.43<br>0.199	[-3.62,0.75]	0.22<br>0.788	[-1.39,1.83]
**Average**	0 [ref]		0 [ref]	
**Below Average**	-1.54<br>0.345	[-4.75,1.66]	0.68<br>0.575	[-1.69,3.04]
**Persistently Low**	1.05<br>0.788	[-6.63,8.74]	1.8<br>0.532	[-3.86,7.47]
**Male**	10.62<br><0.001	[8.55,12.69]	0.06<br>0.943	[-1.47,1.58]
**BMI (age 8)**	0.39<br>0.094	[-0.07,0.84]	0.09<br>0.613	[-0.25,0.42]
**Maternal smoking (recruitment)**	0.29<br>0.874	[-3.24,3.81]	1.25<br>0.345	[-1.35,3.85]

Table S9 continue

b) Lung function trajectories included as continuous predictor

	Systolic (mmHg)		Diastolic (mmHg)	
	Mean<br>p-value	95% CI	Mean<br>p-value	95% CI
	**CRUDE N=492**			
FEV1/FVC (per-class)	2.05<br>**0.005**	[0.63,3.47]	0.15<br>0.754	[-0.80,1.11]
	**ADJUSTED by gender, BMI at 8 years and maternal smoking N=476**			
FEV1/FVC (per-class)	0.34<br>0.638	[-1.07,1.74]	0.08<br>0.887	[-0.96,1.11]
Male	10.57<br><0.001	[8.50,12.64]	0.14<br>0.853	[-1.38,1.67]
BMI (age 8)	0.37<br>0.107	[-0.08,0.83]	0.096<br>0.570	[-0.24,0.43]
Maternal smoking (recruitment)	0.40<br>0.824	[-3.12,3.92]	1.25<br>0.346	[-1.35,3.84]

Note: ‘per-class increase’ is equivalent to ‘with decreasing lung function’

Table S10. *Associations between FEV1/FVC trajectories 8 to 24 years and Cardiovascular Outcomes at 24 years in ALSPAC. Linear regression crude and adjusted analyses weighted by class membership probabilities.*

FEV1/FVC trajectories 8 to 24 years	N (%)	Left Ventricular mass indexed to height 2.7 (g/m^2.7)	Left atrium diameter indexed to height (cm/m)	Left Ventricle Posterior Wall Diastolic Thickness Average (cm)	Left Ventricle Posterior Wall Systolic Thickness Average (cm)	Relative Wall Thicknessɫ
		Mean Difference (95%CI) p-value				
	**CRUDE N=1,660**					
Above Average	862 (52.0)	-1.413 (-2.103, -0.724) <0.001	-3.113 (-4.662, -1.564) <0.001	-0.029 (-0.043, -0.016) <0.001	-0.031 (-0.049, -0.012) 0.001	-0.006 (-0.012, 0.001) 0.09
Average	595 (35.8)	0 [ref]	0 [ref]	0 [ref]	0 [ref]	0 [ref]
Below Average	182 (11.0)	0.486 (-0.609, 1.582) 0.384	2.393 (-0.068, 4.855) 0.057	0.017 (-0.004, 0.038) 0.118	0.037 (0.008, 0.066) 0.014	0.001 (-0.009, 0.011) 0.81
Persistent Low	22 (1.3)	1.891 (-0.904, 4.686) 0.185	5.659 (-0.619, 11.937) 0.077	0.027 (-0.028, 0.081) 0.341	0.060 (-0.014, 0.134) 0.11	-0.005 (-0.030, 0.021) 0.71
	**ADJUSTED by gender, maternal lower education level* and child's BMI at 7 years N=1,460**					
Above Average	753 (51.6)	-0.791 (-1.491, -0.091) 0.027	-1.475 (-2.971, 0.020) 0.053	-0.014 (-0.028, -0.001) 0.039	-0.010 (-0.028, 0.009) 0.296	-0.002 (-0.009, 0.004) 0.47
Average	522 (35.7)	0 [ref]	0 [ref]	0 [ref]	0 [ref]	0 [ref]
Below Average	166 (11.4)	0.254 (-0.836, 1.344) 0.648	1.094 (-1.236, 3.423) 0.357	0.009 (-0.012, 0.030) 0.409	0.023 (-0.005, 0.051) 0.113	0.002 (-0.009, 0.012) 0.78
Persistent Low	20 (1.4)	0.692 (-2.088, 3.473) 0.625	1.334 (-4.606, 7.273) 0.66	-0.003 (-0.057, 0.050) 0.901	0.022 (-0.049, 0.094) 0.536	-0.006 (-0.033, 0.021) 0.65

Granell R, *et al. Thorax* 2024; 79:770–777. doi: 10.1136/thorax-2023-220485

Table S10 continue

FEV1/FVC trajectories 8 to 24 years		Pulse presure (mmHg)		Right carotid intima-media thickness-Mean (mm)	Left carotid intima-media thickness-Mean (mm)	Average carotid intima-media thickness-Mean (mm)
	N (%)	Mean Difference (95% CI) p-value	N (%)		Mean Difference (95% CI) p-value	
	**CRUDE N=3,201**		**CRUDE N=1,710**			**CRUDE N=1,651**
Persistent High	1591 (49.7)	-1.086 (-1.759, -0.413) 0.002	863 (50.5)	-0.005 (-0.011, 0.001) 0.079	-0.003 (-0.009, 0.003) 0.275	-0.004 (-0.009, 0.001) 0.112
Average	1191 (37.2)	0 [ref]	634 (37.0)	0 [ref]	0 [ref]	0 [ref]
Below Average	374 (11.7)	1.225 (0.184, 2.267) 0.021	190 (11.1)	0.006 (-0.004, 0.016) 0.217	0.006 (-0.003, 0.015) 0.206	0.006 (-0.002, 0.014) 0.126
Persistent Low	47 (1.5)	1.958 (-0.666, 4.581) 0.144	24 (1.4)	0.015 (-0.009, 0.039) 0.213	0.009 (-0.015, 0.033) 0.46	0.015 (-0.006, 0.035) 0.156
	**ADJUSTED by gender, maternal lower education level* and child's BMI at 7 years**					
	**N=2,759**		**N=1,502**			**N=1,452**
Persistent High	1364 (49.4)	0.022 (-0.592, 0.636) 0.944	754 (50.1)	-0.003 (-0.009, 0.004) 0.386	-0.002 (-0.008, 0.005) 0.642	-0.002 (-0.007, 0.003) 0.45
Average	1032 (37.4)	0 [ref]	556 (37.0)	0 [ref]	0 [ref]	0 [ref]
Below Average	321 (11.6)	0.219 (-0.728, 1.165) 0.651	172 (11.4)	0.003 (-0.007, 0.013) 0.511	0.003 (-0.006, 0.013) 0.488	0.004 (-0.005, 0.012) 0.398
Persistent Low	43 (1.6)	-0.681 (-2.992, 1.630) 0.563	22 (1.4)	0.012 (-0.013, 0.037) 0.337	-0.003 (-0.028, 0.023) 0.846	0.008 (-0.014, 0.029) 0.491

ɫ 2 × Left Ventricle Posterior Wall Diastolic Thickness Average)/ LV internal diameter diastolic

* Educated to the General Certificate of Education level (school-leaving certificate) or lower.

Table S11. *Associations between FEV1/FVC trajectories 8 to 24 years and Blood Measures at 24 years in ALSPAC. Linear regression crude and adjusted analyses weighted by class membership probabilities.*

FEV1/FVC trajectories 8 to 24 years	N (%)	Average seated systolic blood pressure (mmHg)<br>Mean Difference (95%CI) p-value	Average seated diastolic blood pressure (mmHg)<br>Mean Difference (95%CI) p-value	Heart rate (bpm)<br>Mean Difference (95%CI) p-value
**Crude N=3,201**				
Above Average	1591 (49.7)	-1.812 (-2.666, -0.959) <0.001	-0.726 (-1.320, -0.133) 0.017	0.648 (-0.117, 1.413) 0.097
Average	1191 (37.2)	0 [ref]	0 [ref]	0 [ref]
Below Average	374 (11.7)	1.150 (-0.171, 2.471) 0.088	-0.075 (-0.995, 0.844) 0.872	-0.892 (-2.076, 0.291) 0.14
Persistent Low	47 (1.5)	2.021 (-1.306, 5.348) 0.234	0.063 (-2.252, 2.378) 0.957	-2.142 (-5.123, 0.839) 0.159
**ADJUSTED by gender, maternal lower education level* and child's BMI at 7 years N=2,759**				
Above Average	1364 (49.4)	-0.283 (-1.088, 0.521) 0.49	-0.305 (-0.943, 0.332) 0.348	0.368 (-0.443, 1.178) 0.374
Average	1032 (37.4)	0 [ref]	0 [ref]	0 [ref]
Below Average	321 (11.6)	-0.025 (-1.264, 1.214) 0.968	-0.244 (-1.226, 0.739) 0.627	-0.525 (-1.773, 0.723) 0.41
Persistent Low	43 (1.6)	-1.276 (-4.302, 1.750) 0.408	-0.595 (-2.994, 1.804) 0.627	-1.869 (-4.918, 1.179) 0.229

* Educated to the General Certificate of Education level (school-leaving certificate) or lower.

Table S12. *Associations between FEV1/FVC trajectories 8 to 24 years and Fasting Lipids at 24 years in ALSPAC. Linear regression crude and adjusted analyses weighted by class membership probabilities.*

FEV1/FVC trajectories 8 to 24 years	N (%)	Glucose (mmol/L)	C-Reactive Protein (mg/L)	Triglycerides (mmol/L)	High-density lipoprotein (HDL) (mmol/L)	Low-density lipoprotein (LDL) (mmol/L)	Cholesterol (mmol/L)
		Mean Difference (95%CI) of log-transformed outcome p-value			Mean Difference (95% CI) p-value		
	**Crude N=2,617**						
Above Average	1283 (49.0)	-0.007 (-0.016, 0.002) 0.129	-0.039 (-0.142, 0.064) 0.46	-0.035 (-0.071, 0.000) 0.051	0.054 (0.019, 0.089) 0.003	-0.028 (-0.091, 0.035) 0.381	0.007 (-0.062, 0.076) 0.849
Average	974 (37.2)	0 [ref]	0 [ref]	0 [ref]	0 [ref]	0 [ref]	0 [ref]
Below Average	322 (12.3)	0.004 (-0.009, 0.018) 0.538	-0.139 (-0.296, 0.018) 0.083	0.021 (-0.032, 0.075) 0.436	0.009 (-0.044, 0.062) 0.744	-0.024 (-0.119, 0.071) 0.615	-0.000 (-0.105, 0.104) 0.995
Persistent Low	40 (1.5)	-0.005 (-0.039, 0.029) 0.776	-0.048 (-0.437, 0.341) 0.809	0.121 (-0.015, 0.257) 0.081	-0.080 (-0.213, 0.053) 0.24	0.259 (0.020, 0.499) 0.034	0.231 (-0.033, 0.495) 0.087
	**ADJUSTED by gender, maternal lower education level* and child's BMI at 7 years N=2,269**						
Above Average	1106 (48.7)	0.001 (-0.008, 0.010) 0.822	-0.044 (-0.153, 0.064) 0.423	-0.013 (-0.050, 0.025) 0.512	0.023 (-0.013, 0.059) 0.212	-0.011 (-0.079, 0.056) 0.747	0.004 (-0.070, 0.079) 0.91
Average	847 (37.3)	0 [ref]	0 [ref]	0 [ref]	0 [ref]	0 [ref]	0 [ref]
Below Average	281 (12.4)	-0.003 (-0.016, 0.010) 0.678	-0.117 (-0.281, 0.046) 0.159	0.010 (-0.046, 0.067) 0.719	0.027 (-0.027, 0.081) 0.335	-0.046 (-0.148, 0.055) 0.369	-0.010 (-0.122, 0.101) 0.856
Persistent Low	36 (1.6)	-0.016 (-0.048, 0.017) 0.352	0.030 (-0.368, 0.428) 0.883	0.088 (-0.051, 0.228) 0.215	-0.014 (-0.147, 0.120) 0.84	0.220 (-0.030, 0.471) 0.085	0.242 (-0.034, 0.518) 0.086

* Educated to the General Certificate of Education level (school-leaving certificate) or lower

Table S13. *Associations between FEV₁/FVC trajectories (8 to 24 years) and markers of cardiovascular disease risk at 24 years in ALSPAC stratified by sex.*

	Per-FEV_1/FVC trajectory 8 to 24 years							
MALES ONLY	**N**	**Mean 95% CI**	**P-value**	**Adjusted†**	**P-value**	**N**	**Further Adjusted††**	**P-value**
**Cardiovascular Outcomes at 24 years**								
Left Ventricular Mass Indexed to Height 2.7 (g/$m^{2.7}$)	563	0.80 (0.08, 1.51)	0.029	0.52 (-0.16,1.20)	0.13	501	0.79 (0.08, 1.50)	0.030
Left Ventricle Posterior Wall Systolic Thickness Average (cm)	553	0.02 (0.00, 0.04)	0.021	0.02 (-0.00,0.04)	0.06	491	0.02 (-0.00, 0.04)	0.08
Carotid Femoral Pulse Wave Velocity (m/s)	662	-0.03 (-0.14, 0.08)	0.57	-0.03 (-0.14,0.08)	0.58	592	-0.02 (-0.14, 0.10)	0.69
Pulse Pressure (mmHg)	1097	-0.05 (-0.71, 0.60)	0.87	-0.08 (-0.73,0.58)	0.82	964	-0.13 (-0.83, 0.57)	0.71
Average Carotid Intima-Media Thickness-Mean (mm)	565	0.00 (-0.00, 0.01)	0.10	0.00 (-0.00, 0.01)	0.14	504	0.01 (-0.00, 0.01)	0.06
**Blood Pressure Measures at 24 years**								
Systolic (mmHg)	1097	-0.06 (-0.86, 0.74)	0.89	-0.16 (-0.96, 0.64)	0.69	964	-0.26 (-1.10, 0.59)	0.55
Diastolic (mmHg)	1097	-0.01 (-0.62, 0.61)	0.98	-0.09 (-0.70, 0.53)	0.78	964	-0.13 (-0.77, 0.52)	0.71
**Fasting Lipids at 24 years**								
Triglycerides (mmol/L, log)	964	0.03 (-0.01, 0.06)	0.14	0.03 (-0.01, 0.06)	0.16	855	0.02 (-0.01, 0.06)	0.21
HDL (mmol/L)	964	0.01 (-0.02, 0.04)	0.66	0.00 (-0.02, 0.03)	0.74	855	0.00 (-0.03, 0.03)	0.84

Table S13 continue

	Per-FEV_1/FVC trajectory 8 to 24 years							
FEMALES ONLY	**N**	**Mean 95%CI**	**P-value**	**Adjusted†**	**P-value**	**N**	**Further Adjusted††**	**P-value**
**Cardiovascular Outcomes at 24 years**								
Left Ventricular Mass Indexed to Height 2.7 ($g/m^{2.7}$)	896	0.83 (0.24, 1.42)	0.006	0.61 (0.03,1.19)	0.038	785	0.59 (-0.02, 1.21)	0.06
Left Ventricle Posterior Wall Systolic Thickness Average (cm)	868	0.02 (0.00, 0.03)	0.043	0.01 (-0.00,0.03)	0.18	760	0.01 (-0.00, 0.03)	0.17
Carotid Femoral Pulse Wave Velocity (m/s)	1039	0.01 (-0.07, 0.10)	0.80	0.01 (-0.08,0.09)	0.91	906	-0.00 (-0.09, 0.09)	0.94
Pulse Pressure (mmHg)	1661	0.16 (-0.30, 0.62)	0.49	0.07 (-0.39,0.53)	0.77	1436	0.20 (-0.30, 0.70)	0.44
Average Carotid Intima-Media Thickness-Mean (mm)	885	0.00 (-0.00, 0.01)	0.35	0.00 (-0.00, 0.01)	0.48	772	0.00 (-0.00, 0.01)	0.39
**Blood Pressure Measures at 24 years**								
Systolic (mmHg)	1661	0.54 (-0.12, 1.20)	0.11	0.26 (-0.39, 0.91)	0.44	1436	0.46 (-0.26, 1.17)	0.21
Diastolic (mmHg)	1661	0.38 (-0.15, 0.91)	0.16	0.19 (-0.34, 0.71)	0.49	1436	0.26 (-0.32, 0.83)	0.39
**Fasting Lipids at 24 years**								
Triglycerides (mmol/L, log)	1304	0.01 (-0.02, 0.04)	0.37	0.01 (-0.02, 0.04)	0.64	1127	0.00 (-0.03, 0.04)	0.84
HDL (mmol/L)	1304	-0.02 (-0.06, 0.01)	0.18	-0.02 (-0.05, 0.02)	0.33	1127	-0.02 (-0.06, 0.02)	0.29

† Adjusted by maternal lower education level (educated to the General Certificate of Education level 'school-leaving certificate' or lower) and child's BMI at 7 year. †† Further Adjusted by low birth weight, maternal postnatal smoking and child tobacco smoke exposure at 8 years.

Table S14. *Associations between* ***predicted FEV_1/FVC trajectories*** *(8 to 24 years) and markers of cardiovascular disease risk at 24 years in ALSPAC*

	N	Mean 95%CI per-FEV_1/FVC trajectory 8 to 24 years CRUDE effect	P-value	N	Mean 95%CI **per-predicted** FEV_1/FVC trajectory 8 to 24 years CRUDE effect	P-value
Cardiovascular Outcomes at 24 years						
Left Ventricular Mass Indexed to Height 2.7 ($g/m^{2.7}$)	1460	1.14 (0.68, 1.60)	1.30×10^{-6}	1501	0.90 (0.43, 1.36)	1.5×10^{-4}
Left Ventricle Posterior Wall Systolic Thickness Average (cm)	1422	0.033 (0.020, 0.045)	2.0×10^{-7}	1464	0.02 (0.01, 0.03)	0.004
Carotid Femoral Pulse Wave Velocity (m/s)	1702	0.058 (-0.011, 0.13)	0.10	1746	-0.01 (-0.08, 0.06)	0.70
Pulse Pressure (mmHg)	2759	1.10 (0.65, 1.55)	1.82×10^{-6}	2819	0.02 (-0.44, 0.47)	0.94
Average Carotid Intima-Media Thickness-Mean (mm)	1451	0.005 (0.001, 0.008)	0.008	1487	0.00 (-0.00, 0.01)	0.074
Blood Pressure Measures at 24 years						
Systolic (mmHg)	2759	1.44 (0.87, 2.02)	8.4×10^{-7}	2819	0.18 (-0.40, 0.75)	0.55
Diastolic (mmHg)	2759	0.34 (-0.06, 0.74)	0.09	2819	0.16 (-0.24, 0.56)	0.43
Fasting Lipids at 24 years						
Triglycerides (mmol/L, log)	2269	0.032 (0.009, 0.056)	0.006	2311	0.02 (-0.01, 0.04)	0.16
HDL (mmol/L)	2269	-0.035 (-0.059, -0.012)	0.003	2311	-0.01 (-0.03, 0.01)	0.45

SUPPLEMENTARY REFERENCES

1. Belgrave DCM, Granell R, Turner SW, et al. Lung function trajectories from pre-school age to adulthood and their associations with early life factors: a retrospective analysis of three population-based birth cohort studies. *Lancet Respir Med* 2018; **6**(7): 526-34.
2. Berry CE, Billheimer D, Jenkins IC, et al. A distinct low lung function trajectory from childhood to the fourth decade of life. *American journal of respiratory and critical care medicine* 2016; **194**(5): 607-12.
3. Karmaus W, Mukherjee N, Janjanam VD, et al. Distinctive lung function trajectories from age 10 to 26 years in men and women and associated early life risk factors - a birth cohort study. *Respir Res* 2019; **20**(1): 98.
4. McGeachie MJ, Yates KP, Zhou X, et al. Patterns of growth and decline in lung function in persistent childhood asthma. *New England Journal of Medicine* 2016; **374**(19): 1842-52.
5. Sanna F, Locatelli F, Sly PD, et al. Characterisation of lung function trajectories and associated early-life predictors in an Australian birth cohort study. *ERJ Open Res* 2022; **8**(1).
6. Weber P, Menezes AMB, Goncalves H, et al. Characterisation of pulmonary function trajectories: results from a Brazilian cohort. *ERJ Open Res* 2020; **6**(3).
7. Bui DS, Lodge CJ, Burgess JA, et al. Childhood predictors of lung function trajectories and future COPD risk: a prospective cohort study from the first to the sixth decade of life. *The Lancet Respiratory Medicine* 2018.
8. Boyd A, Golding J, Macleod J, Lawlor DA, Fraser A, Henderson J, Molloy L, Ness A, Ring S, Davey Smith G. Cohort Profile: The 'Children of the 90s'--the index offspring of the Avon Longitudinal Study of Parents and Children. *Int J Epidemiol* 2013; **42**(1): 111-27.
9. Fraser A, Macdonald-Wallis C, Tilling K, et al. Cohort Profile: the Avon Longitudinal Study of Parents and Children: ALSPAC mothers cohort. *Int J Epidemiol* 2013; **42**(1): 97-110.
10. Northstone K, Lewcock M, Groom A, et al. The Avon Longitudinal Study of Parents and Children (ALSPAC): an update on the enrolled sample of index children in 2019. *Wellcome Open Res* 2019; **4**: 51.
11. Harris PA, Taylor R, Thielke R, Payne J, Gonzalez N, Conde JG. Research electronic data capture (REDCap)--a metadata-driven methodology and workflow process for providing translational research informatics support. *J Biomed Inform* 2009; **42**(2): 377-81.
12. Custovic A, Simpson BM, Murray CS, et al. The National Asthma Campaign Manchester Asthma and Allergy Study. *Pediatr Allergy Immunol* 2002; **13**(s15): 32-7.
13. Kurukulaaratchy RJ, Fenn M, Twiselton R, Matthews S, Arshad SH. The prevalence of asthma and wheezing illnesses amongst 10-year-old schoolchildren. *Respir Med* 2002; **96**(3): 163-9.
14. Kurukulaaratchy RJ, Fenn MH, Waterhouse LM, Matthews SM, Holgate ST, Arshad SH. Characterization of wheezing phenotypes in the first 10 years of life. *Clin Exp Allergy* 2003; **33**(5): 573-8.
15. Arshad SH, Holloway JW, Karmaus W, et al. Cohort Profile: The Isle Of Wight Whole Population Birth Cohort (IOWBC). *Int J Epidemiol* 2018; **47**(4): 1043-4i.
16. Miller MR, Hankinson J, Brusasco V, et al. Standardisation of spirometry. *European respiratory journal* 2005; **26**(2): 319-38.
17. Beydon N, Davis SD, Lombardi E, et al. An official American Thoracic Society/European Respiratory Society statement: pulmonary function testing in preschool children. *Am J Respir Crit Care Med* 2007; **175**(12): 1304-45.
18. Timpka S, Macdonald-Wallis C, Hughes AD, et al. Hypertensive Disorders of Pregnancy and Offspring Cardiac Structure and Function in Adolescence. *J Am Heart Assoc* 2016; **5**(11).
19. Kracht D, Shroff R, Baig S, et al. Validating a new oscillometric device for aortic pulse wave velocity measurements in children and adolescents. *Am J Hypertens* 2011; **24**(12): 1294-9.

20. Lodrup Carlsen KC, Roll S, Carlsen KH, et al. Does pet ownership in infancy lead to asthma or allergy at school age? Pooled analysis of individual participant data from 11 European birth cohorts. *PLoS One* 2012; **7**(8): e43214.
21. Roberts G, Peckitt C, Northstone K, et al. Relationship between aeroallergen and food allergen sensitization in childhood. *Clin Exp Allergy* 2005; **35**(7): 933-40.
22. Semic-Jusufagic A, Belgrave D, Pickles A, et al. Assessing the association of early life antibiotic prescription with asthma exacerbations, impaired antiviral immunity, and genetic variants in 17q21: a population-based birth cohort study. *Lancet Respir Med* 2014; **2**(8): 621-30.
23. Haider S, Granell R, Curtin J, et al. Modeling Wheezing Spells Identifies Phenotypes with Different Outcomes and Genetic Associates. *Am J Respir Crit Care Med* 2022; **205**(8): 883-93.
24. Oberski D. Latent Profile and Latent Class Analysis. Modern Statistical Methods for HCI: Springer, Cham; 2016: 275-87.
